# Intrathecal Human Tetanus Immunoglobulin for Post-Neonatal Tetanus in Resource-Limited Settings: An Old Idea with New Evidence

**DOI:** 10.4269/ajtmh.25-0768

**Published:** 2026-01-27

**Authors:** Samriti Gupta, Abhijit Choudhary

**Affiliations:** ^1^ All India Institute of Medical Sciences, Vijaypur, Jammu, India;; ^2^ All India Institute of Medical Sciences, Nagpur, Maharashtra, India

Tetanus remains one of the most preventable, yet persistent, causes of childhood mortality worldwide. Despite the availability of effective vaccines for decades, tetanus continues to pose a threat in regions with limited healthcare access, suboptimal immunization coverage, and poor wound-care practices. Globally, tetanus incidence declined from an estimated 308,931 cases in 1990 to 17,788 in 2021, with mortality falling from 24.6 to 1.3 deaths per 100,000 children.^1^^,^^2^ These gains have been most pronounced among pregnant women and neonates, culminating in World Health Organisation (WHO)-led guidelines on maternal and neonatal tetanus elimination in 2018.^3^ Nevertheless, the disease burden remains substantial in low- and middle-income countries (LMICs), largely due to inadequate vaccination and restricted access to timely care. Post-neonatal tetanus, though less frequent than neonatal disease, continues to cause significant morbidity and mortality, reflecting persistent immunization gaps, poor primary wound management, and delayed treatment seeking.^1^^,^^2^^,^^4^

Management of established tetanus is based on five pillars: 1) source control with wound debridement and antibiotics; 2) neutralization of unbound toxin, traditionally with intramuscular (IM) human tetanus immunoglobulin (TIG); 3) control of muscle spasms; 4) comprehensive organ support, including mechanical ventilation and vasoactive agents; and 5) treatment of autonomic instability.^4^^,^^5^ In severe post-neonatal tetanus, intrathecal human TIG (ITIG) is an alternative approach, delivering immunoglobulin directly into cerebrospinal fluid to potentially achieve faster central toxin neutralization than with intramuscular administration.^4^^,^^6^

The evidence supporting use of ITIG remains heterogeneous, derived from small trials conducted in premodern intensive care settings with limited reporting of pediatric outcomes, resulting in wide global variation in clinical practice. A 2006 meta-analysis of randomized controlled trials (RCTs) involving 942 patients, including adults and neonates, demonstrated a significant mortality benefit for intrathecal TIG or anti-tetanus serum compared with IM therapy alone (relative risk [RR] = 0.71; 95% CI = 0.62–0.81). However, these pooled data included older trials, and their relevance has been questioned in light of more recent, higher-quality studies showing limited benefit.^7^ A large, blinded 2 × 2 factorial RCT conducted in adults in Vietnam rigorously evaluated the addition of ITIG to standard care (IM TIG) and found no clear benefit in major outcomes, including the need for mechanical ventilation (43% with vs 50% without ITIG, RR = 0.87, 95% CI = 0.66–1.13; *P*-value = 0.29).^8^ In contrast, smaller randomized and observational studies from India in adults have suggested potential benefits, such as reduced disease severity, shorter illness duration, and faster recovery, although these findings are constrained by limited sample sizes. A double-blind, sham-controlled trial including 36 adults reported reduced morbidity, disease progression, and mortality (20% vs 30%) with ITIG in patients with mild tetanus.^9^ A later Indian RCT comparing intrathecal therapy alone with combined intrathecal and IM TIG in 125 patients found no mortality difference, but demonstrated faster recovery and shorter hospital stay among survivors receiving combined therapy.^10^

Pediatric data, largely retrospective, appear more supportive of ITIG. Studies from India reported substantial reductions in morbidity and mortality, particularly in mild-to-moderate tetanus.^11^^,^^12^ A series of 41 children observed low mortality following ITIG use (7.3%),^11^ while a larger cohort of 100 neonatal and post-neonatal cases showed significantly better survival with combined intrathecal and IM TIG compared with IM treatment alone (95.3% vs 75%).^12^ Despite these encouraging regional findings, the WHO continues to recommend IM TIG as standard care, citing inconsistent evidence for routine intrathecal administration, which remains limited to specialized centers or clinical trials.

A new paper by Jayaram and colleagues in the 114-3 March 2026 issue of the *American Journal of Tropical Medicine and Hygiene* presents a 19-year, single-center retrospective comparative analysis of 63 children with post-neonatal tetanus cared for in a tertiary pediatric intensive care unit (PICU), comparing outcomes before and after routine adoption of ITIG.^13^ The authors report a striking association between ITIG use and reduction of in-hospital mortality between groups with and without ITIG use (10% vs 50%). There were also reduced rates of severe complications, including rhabdomyolysis, shock, and acute kidney injury. However, the durations of mechanical ventilation and PICU stay among survivors did not differ between the groups. These findings add to a small but growing pediatric literature suggesting substantial clinical benefit from ITIG in children with severe tetanus.

The current study represents one of the largest pediatric cohorts evaluating the clinical impact of ITIG and provides valuable real-world evidence from an LMIC country tertiary-care setting over a 19-year period. The detailed documentation of baseline disease severity, organ-support requirements, and major complications strengthens the clinical relevance and external validity of the findings.^13^

The marked reduction in in-hospital mortality associated with ITIG in the new study is noteworthy, but should be interpreted with caution given its retrospective, nonrandomized design. ITIG was adopted as routine practice after 2012, introducing potential temporal confounding. Improvements in PICU care over time, including improved staffing, enhanced infection control, and advances in supportive management such as ventilation strategies, may have independently contributed to better outcomes in the later period. The authors accounted for baseline severity and performed multivariate regression, identifying the absence of ITIG as an independent predictor of mortality alongside established risk factors such as shock and autonomic dysfunction. However, residual confounding cannot be excluded. Unmeasured factors may have included changes in referral patterns, evolution in ventilation practices, and improvements in nursing and medical care. Importantly, the lack of significant differences in duration of mechanical ventilation and PICU length of stay among survivors suggests that ITIG may primarily reduce mortality in the most critically ill children, without much benefit in reducing morbidity. Although ITIG was given within 24 hours of admission, delays in presentation to the tertiary referral center may have resulted in administration of ITIG in the late phase of illness. Previous studies suggested that earlier administration in the course of illness was more beneficial, but this was not observed in the new study despite a significant difference in mortality.^9^^,^^11^^,^^13^

Severe pediatric tetanus continues to carry high mortality across LMIC countries. In experienced centers, ITIG is relatively low cost and technically feasible. This study supports considering ITIG as an adjunctive therapy for severe post-neonatal tetanus where lumbar puncture, strict sterile technique, access to human TIG, and high-quality intensive care are assured. Careful local risk–benefit assessment remains essential. Definitive, large RCTs are needed, especially in LMIC settings, to determine the effectiveness of ITIG, focusing on early use, optimal dosing, and outcomes in neonates, children, and adults. Meanwhile, adoption of ITIG use may be instituted along with strengthening vaccination programs globally, as prevention remains the most effective strategy for eliminating this devastating disease.
